# The Potential Use of Herbal Fingerprints by Means of HPLC and TLC for Characterization and Identification of Herbal Extracts and the Distinction of Latvian Native Medicinal Plants

**DOI:** 10.3390/molecules27082555

**Published:** 2022-04-15

**Authors:** Ance Bārzdiņa, Artūrs Paulausks, Dace Bandere, Agnese Brangule

**Affiliations:** 1 Department of Pharmaceutical Chemistry, Riga Stradiņš University, 16 Dzirciema Str., LV-1007 Riga, Latvia; arturs.paulausks@rsu.lv (A.P.); dace.bandere@rsu.lv (D.B.); agnese.brangule@rsu.lv (A.B.); 2 Baltic Biomaterials Centre of Excellence, Riga Technical University, 1 Kalku Str., LV-1658 Riga, Latvia

**Keywords:** herbal medicine, fingerprinting, HPLC, TLC, chemometrics, multivariate analysis

## Abstract

The growing market of herbal medicines, the increase in international trade in Latvia, and the lack of adequate analytical methods have raised the question of the potential use of herbal fingerprinting methods. In this study, high-performance liquid chromatography (HPLC) and thin layer chromatography (TLC) methods were developed for obtaining chromatographic fingerprints of four taxonomically and evolutionary different medicinal plants (*Hibiscus sabdariffa* L., *Calendula officinalis* L., *Matricaria recutita* L., *Achillea millefolium* L.). Retention time shifting, principal component analysis (PCA), hierarchical cluster analysis (HCA), and orthogonal projections to latent structures (OPLS) analysis were used to improve and analyze the obtained fingerprints. HPLC data detection at 270 nm was determined superior to 360 nm for the distinction of medicinal plants and used data alignment method significantly increased similarity between samples. Analyzed medicinal plant extracts formed separate, compact clusters in PCA, and the results of HCA correlated with the evolutionary relationships of the analyzed medicinal plants. Herbal fingerprinting using chromatographic analysis coupled with multivariate analysis has a great potential for the identification of medicinal plants as well as for the distinction of Latvian native medicinal plants.

## 1. Introduction

Although in the last hundred years the backbone of Western medicine has been a great diversity of chemically synthesized drugs, billions of people still use herbal medicine for its well-known wellness-inducing properties and as a prophylactic and therapeutic tool for both minor and chronic health problems [1,2,3]. With an increase in vegetarian and vegan diets, the promotion of healthier lifestyles, and high prices of medication, herbal medicines have become a top choice for many consumers and have shown steady market growth in the 21st century, with the market size of global botanical supplements being valued at USD 27.47 billion in 2020 and the revenue forecast for 2028 being USD 55.18 billion [2,4,5].

The chemical composition of medicinal plants is very complex, with hundreds or even thousands of chemical components, which can be affected by such factors as growth and storage conditions, genetic composition, soil components, time of harvest, processing methods, and others [6]. All of these factors contribute to the batch-to-batch chemical variability of herbal medicines, supplements, and other products like herbal teas, and play a significant role in the physiological or pharmaceutical activities of these products [1].

Latvians have deep-rooted traditions and knowledge about medicinal plant use that are to this day still put in practice [7]. Many medicinal plants are also cultivated and processed in Latvia for the pharmaceutical and food industry, however with growing demand, the import of medicinal plants from foreign countries has grown from 2.98 million euros in 2015 to 11.99 million euros in 2020 [8]. The increasing international trade of raw herbal materials around the world has raised concern about the possible contamination and adulteration of the materials [9,10,11]. Several studies have reported species adulterations ranging from 21% to even 80% of raw herbal samples, greatly impacting the biological activity and chemical composition of the materials [9,11,12,13]. Therefore, the establishment of authentic botanical species standards and analytical methods that can be used for identity tests is crucial [9].

Pharmacopeias and regulatory instances, like European Pharmacopoeia, mostly suggest that the identification and quantitative assay of herbal medicines should be performed using sensory inspections and a quantitative determination of a few key components of each herb [14,15]. However, sensory inspections can differ between specialists and are not adequately validated, and the conclusions can be subjective [16,17]. Additionally, the synergic effect of multiple components is why the single key component quantitative determination is not sufficient for the quality assessment of herbal medicines [18,19].

The chemical fingerprinting method, approved by the World Health Organization (WHO), the Food and Drug Administration (FDA) of the USA, the European Medicines Agency (EMA), and others, can provide a comprehensive chemical description of herbal medicines [4,20,21]. The characteristic profiles and patterns reflecting the complex chemical composition of herbal samples, called fingerprints, can be established using multiple techniques, both chromatographic and spectroscopic [1,22]. There has been previous research on spectroscopic fingerprints of Latvian native medicinal plants, but no studies on Latvian native medicinal plant chromatographic fingerprints have been conducted to this day. Therefore, this study focuses on high-performance liquid chromatography, equipped with a UV detector (HPLC-UV) and thin layer chromatography (TLC) methods [23,24]. Liquid chromatography has been the most popular herbal fingerprinting method because of the advantages such as wide suitability, high resolution, selectivity, sensitivity, reproducibility, and a fully automatable operation [15,25,26]. TLC has also been consistently used in the analysis of herbal medicines, as it is one of the analytical methods provided by European Pharmacopeia, but with the advances in TLC equipment and automatization, this method has also found a place in the scope of chromatographical fingerprinting, because of its simplicity, versatility, specific sensitivity, high throughput, and simple sample preparation [27,28]. 

Since chromatography methods provide very large the data amounts, multivariate statistical analysis methods can be used to reduce the large amount of data and extract information and characters of interest [29]. Methods of multivariate statistical analysis can be grouped according to their purposes, such as unsupervised or supervised. Unsupervised methods such as principal component analysis (PCA) and hierarchical clustering analysis (HCA) are the most widely used for the analysis of herbal medicines, since these methods do not require a dependent variable for modeling [1,30,31]. PCA describes the correlation between a large number of variables using fewer principal components (PCs) while HCA establishes clusters of data, thereby visualizing the main patterns. PCA and HCA can be used for taxonomic discrimination, quality assessment, and geographic origin determination of medicinal plants [31,32,33]. Orthogonal projection to latent structures (OPLS) analysis is a supervised statistical method suitable for showing differences between two predefined groups or systems [1,34,35]. This method explains which variables have the most significant discriminatory power and how the variables are correlated. This study used OPLS to obtain the optimal separation of herbal extract mixtures. To compare two fingerprints or to evaluate the (dis)similarity of the fingerprints, most often the product-moment or the Pearson’s correlation coefficient *r* (−1 ≤ *r* ≤ 1) is used [36]. In this study Pearson’s correlation coefficient values were calculated and PCA, HCA, and OPLS analysis was performed to analyze the obtained fingerprints. 

The aim of this study was to determine the potential use of herbal fingerprinting by means of high-performance liquid chromatography (HPLC) and thin layer chromatography (TLC) in conjunction with chemometrics for characterization and identification of medicinal plant extracts, as well as to determine if the herbal fingerprinting method can be used for the distinction of Latvian native medicinal plants. Four taxonomically different medicinal plants (*Hibiscus sabdariffa* L., *Calendula officinalis* L., *Matricaria recutita* L., and *Achillea millefolium* L.) were chosen for this study based on their phylogenetic relationships and whether or not the medicinal plant is native to Latvia. Since previous studies have mainly focused on obtaining chromatographic fingerprints of only one genus or species and used vastly different TLC and HPLC method parameters, this study analyzed taxonomically and chemically different medicinal plants and aimed to develop analytical methods with wide suitability that can be used for fingerprinting analysis of several medicinal plants, therefore making the process of identifying medicinal plants cheaper, faster, and more efficient [37,38]. To practically evaluate the application of the herbal fingerprinting method, mixed extracts made from combinations of analyzed medicinal plants were made and the possibility of identifying the components of the mixed extracts was investigated.

## 2. Results and Discussion

### 2.1. Advantages and Challenges of Thin Layer Chromatography 

**Figure 1:**
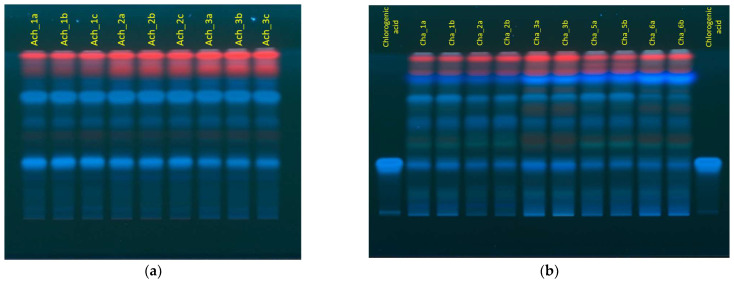
Developed HPTLC plates under 366 nm UV light. (**a**) Developed HPTLC plate of yarrow extracts; (**b**) developed HPTLC plate of chamomile extracts and reference standard—chlorogenic acid.

The efficacy of thin layer chromatography can be greatly influenced by the used mobile phase. We tested a mobile phase consisting of ethyl acetate, water, and formic acid, as it was provided as a method for thin layer chromatography for calendula flowers by European Pharmacopoeia [39]. This mobile phase was efficient in the separation of chemical components of all examined herbal extracts (Figure 1). All photos of developed HPTLC plates under 366 nm UV light can be seen in Figures S1–S4.

Chamber saturation remarkably influences the reproducibility and quality of the results of thin layer chromatography [40]. We found that a 20-min-long chamber saturation is an insufficient time, and the migration of samples isn’t straight, thereby compromising the results. When the chamber saturation was prolonged to 30 min, the migration trajectory greatly improved. The best separation of chemical components was observed when the height of development was 80 mm. As the separated compounds responded to UV light well, derivatization reagents were not applied to the plates.

Chlorogenic acid and rutin were chosen as reference standards, and their R_f_ values were measured. Chlorogenic acid was seen as a blue band under 366 nm UV light with a R_f_ value of 0.33. A similar blue band with the same R_f_ value was seen on plates with extracts of all analyzed medicinal plants, suggesting chlorogenic acid’s presence in all analyzed extracts. Chlorogenic acid was also seen as a clear peak in chromatograms, which were provided by the used software. In previous TLC studies, chlorogenic acid has also been seen as a blue band under 366 nm UV light and detected in calendula, yarrow, and chamomile extracts, thereby the findings of this study coincide with previous research [41,42].

Rutin was seen as a brown band under 366 nm UV light with an R_f_ value of 0.26. A similar color band with the same R_f_ value was observed in samples of calendula extracts, indicating its presence in the above-mentioned extracts. This coincides with research by Agatanovic-Kustrin et al., in which rutin had also been detected in calendula extracts [42]. In our research, rutin, compared to chlorogenic acid, was not seen as a peak in chromatograms provided by the software, indicating a problem with color capture and interpretation of the used software. In addition, bands, which were seen red under 366 nm UV light, were not properly displayed as separate, adequate peaks in chromatograms. This problem significantly impacted data processing and result interpretation. Different TLC image processing methods like splitting a photograph through red, green, and blue channel filters, and denoising, are now being developed to enhance the selectivity and precision of results, but these methods were not tested in this research [43,44].

Since the above-mentioned problem of color capture and interpretation was encountered, the results of TLC were used only as an initial screening tool to characterize the chemical composition of the analyzed herbal extracts. The presence of chlorogenic acid, rutin, and other reference standards in analyzed herbal extracts was later checked using HPLC-UV. In future research, the TLC system could be combined with mass-spectrometry for the identification of separated chemical compounds. 

### 2.2. Method Validation of HPLC Fingerprint Analysis

The HPLC method was validated in terms of system adaptability, intra-day, inter-day precision, and repeatability. System adaptability was assessed by injecting one calendula sample solution six times. The average similarity was 0.9758. To test the reproducibility of this method, three runs of the same extract solution (calendula) and three replications of that medicinal plant sample were analyzed. The average similarity was 0.9944. The intra-day precision test was performed by analysis of the yarrow extracts. Five yarrow extracts were injected three times each on the same day. The average similarity between them was calculated to be 0.9803. The inter-day precision was examined by analyzing two extracts of each medicinal plant in duplicate on three separate days. Retention time shifting was observed, therefore retention times were adjusted using the data alignment method described in Section 2.4. The average similarity between extracts of each medicinal plant was calculated to be 0.9784. Since the results of validation were consistent and adequate, the used HPLC method was determined suitable for obtaining herbal fingerprints.

### 2.3. Obtained Chemical Profiles and Identification of Chemical Compounds Using HPLC

**Figure 2:**
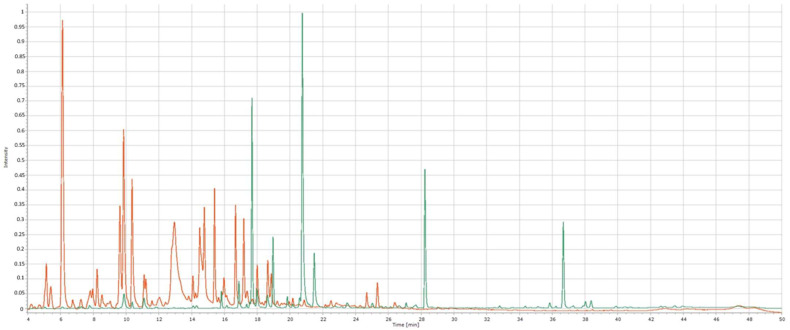
The difference between the chemical profiles of roselle and calendula, HPLC, 270 nm. Orange—chromatogram of roselle extract; green—chromatogram of calendula extract.

The HPLC method provided distinctive chemical profiles, also known as fingerprints, for each of the analyzed medicinal plants. In Figure 2, the clear difference between roselle and calendula herbal fingerprints can be seen, suggesting the contrast of the chemical composition of a Latvian native medicinal plant and an exotic/tropical plant like roselle. 

**Figure 3:**
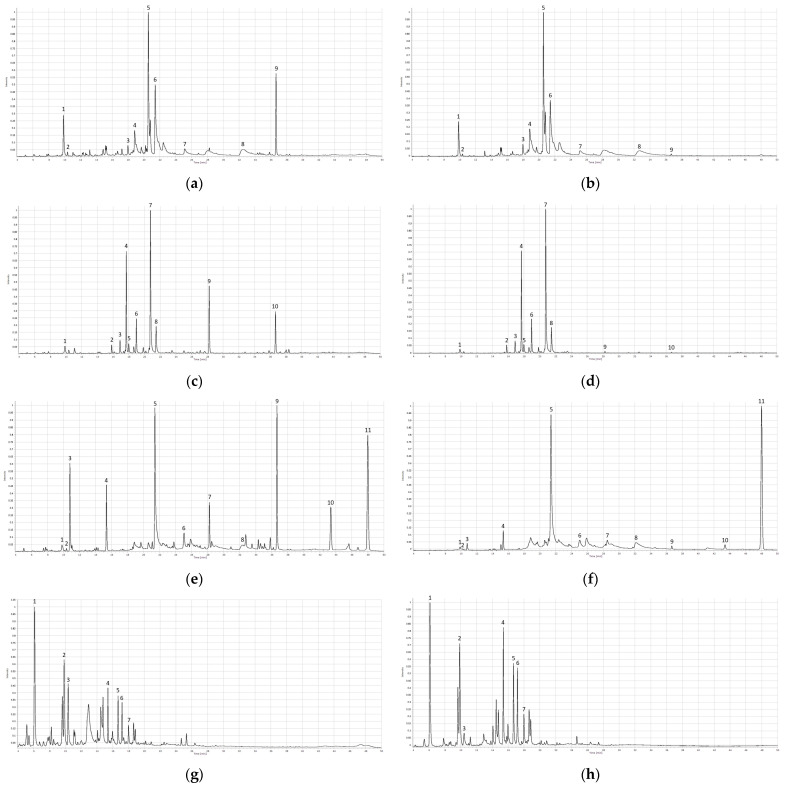
HPLC chromatograms for each medicinal plant. (**a**) Average chromatogram of yarrow extracts, 270 nm; (**b**) average chromatogram of yarrow extracts, 360 nm; (**c**) average chromatogram of calendula extracts, 270 nm; (**d**) average chromatogram of calendula extracts, 360 nm; (**e**) average chromatogram of chamomile extracts, 270 nm; (**f**) average chromatogram of chamomile extracts, 360 nm; (**g**) average chromatogram of roselle extracts, 270 nm; (**h**) average chromatogram of roselle extracts, 360 nm.

Peaks that were visible in all samples at both wavelengths and were of reasonable heights were labeled as “common peaks”. These peaks were numbered based on their elution order: 10 common peaks for calendula, 11—for chamomile, 9—for yarrow, and 7—for roselle were found (Figure 3). Differences in peak intensity at 270 nm or 360 nm wavelengths were observed. For yarrow, chamomile, and calendula common peak intensity was superior at 270 nm compared with 360 nm. The opposite was observed when analyzing roselle samples—peak intensity was higher at 360 nm than at 270 nm, showing analytical differences between Latvian native medicinal plants and roselle. In general, more peaks were visible at 270 nm, thereby providing more detailed chemical profiles and making this method superior for obtaining herbal fingerprints. Detection wavelengths’ influence on peak intensity has been researched by Yang et al. concluding that detection of chlorogenic acid at 273 nm is much more effective than at 360 nm, while the intensity of the rutin peak is higher at 360 nm [45].

As all of the analyzed medicinal plants are considered polyphenol-rich, five well-known polyphenols—chlorogenic acid, caffeic acid, rutin, apigenin, and apigenin-7-glucoside—were chosen as reference standards [46]. To identify some of the common peaks of samples, the retention times (RT) of reference-standard solutions and retention times of common peaks were matched. To verify the findings, a standard adding method was executed. If the results matched, it was assumed that the peak was identified. 

In calendula extracts, 2 common peaks were identified: peak no 1, chlorogenic acid; peak no 5, rutin. In a previous study of calendula extracts, rutin was also identified, but chlorogenic acid had not been detected [47].

In chamomile extracts, 4 common peaks were identified: peak no 1, chlorogenic acid; peak no 2, caffeic acid; peak no 5, apigenin-7-glucoside; peak no 8, apigenin. These findings correlate with previous studies of chamomile, in which these compounds have also been found [48,49,50]. In some studies, rutin had also been found in chamomile extracts, but when analyzing our samples, rutin was not detected [48,50].

In yarrow extracts, 5 common peaks were identified: peak no 1, chlorogenic acid; peak no 2, caffeic acid; peak no 3, rutin; peak no 6, apigenin-7-glucoside; peak no 8, apigenin. All of the above-mentioned compounds, except caffeic acid, have also been identified in previous studies concerning the chemical composition of yarrow [51,52,53]. 

In roselle samples, 3 peaks were identified: peak no 2, chlorogenic acid; peak no 3, caffeic acid; peak no 7, rutin. These substances have also been identified by analyzing roselle extracts in previous studies [54,55,56]. 

Information regarding retention times of the found compounds in this study can be found in Table 1, but obtained average normalized chromatograms with labeled common peaks can be seen in Figure 3 and Figures S5–S12. The HPLC method was effective in separating chemical compounds of all analyzed extracts, but further research must be conducted using mass-spectrometry to verify the identity of found compounds.

### 2.4. The Efficacy of HPLC Data Alignment Optimization

Retention time shifting is a common problem regarding liquid chromatography that can be caused by minor changes in the mobile-phase organic concentration, pH, flow rate, and other factors [57]. In this study, retention time shifting between samples impacted their similarity, which was assessed by calculating Pearson’s correlation coefficient values. To minimize the impact of retention time shifting, retention time adjusting was executed. Several retention time alignment methods have been previously developed, which significantly reduce the influence of retention time shifts, improve similarity between samples, and lead to much more accurate conclusions [50,58,59]. In this study, retention times were adjusted to the average chromatogram that was obtained for each medicinal plant and wavelength, respectively.

Retention time adjustment significantly improved the average Pearson’s correlation coefficient values for all analyzed medicinal plants (Table 2). The HPLC method provided the average Pearson’s correlation coefficient value *r* for raw data to be higher than 0.6805 for all medicinal plants at both wavelengths, while after the retention time adjustment, the average value for chamomile samples was no less than 0.7995, and for other analyzed medicinal plants it was no less than 0.9305. The improvement of Pearson’s correlation coefficient values differed between medicinal plants. If the similarity was high even for raw data, for example—roselle samples at 270 nm, then the increase of *r* values was not so significant. But if the similarity for raw data was lower, for example—yarrow samples, the increase in *r* values after retention time alignment were as high as 36.74% at 270 nm. The lowest of average *r* values after retention time adjustment were for chamomile samples. Raw *Matricaria recutita* L. samples were visually diverse and had the largest sample size, thereby possibly explaining the diversity of chemical composition between samples. In a study by Jiao et al., retention time alignment was applied to fingerprint analysis and also provided satisfactory results [60].

**Figure 4:**
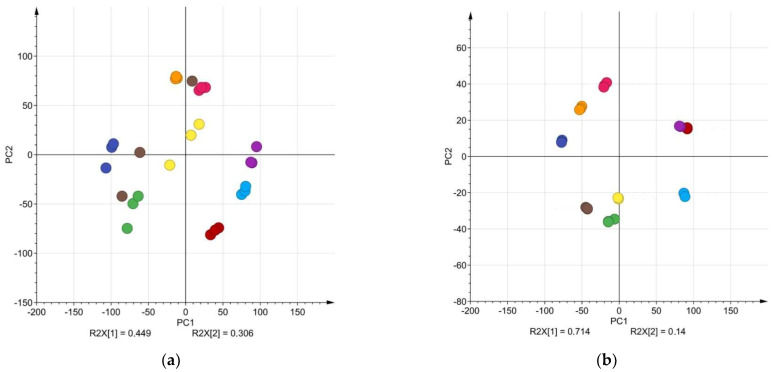
The impact of retention time adjustment to the PCA of *Matricaria recutita* L. HPLC data. (**a**) The PCA clusters for raw chamomile extract HPLC data, 360 nm (extracts made from different *Matricaria recutita* L. samples are shown in different colors, extracts were made in triplicate); (**b**) the PCA clusters for chamomile extract HPLC data with adjusted retention times (extracts made from different *Matricaria recutita* L. samples are shown in different colors, extracts were made in triplicate).

Differences between raw data and adjusted data can also be seen in the PCA of nine chamomile sample triplicates (Figure 4). The PCA of adjusted data resulted in 6 components with R2(X) of 97.9% and Q2 95.9%. After adjusting retention times, the chamomile extracts from each sample formed compact clusters in comparison to raw data, proving the efficacy of the used data alignment method (Figure 4). The PCA analysis was performed using a two-component model with a total variance 75.5% (PC1 44.9%, PC2 30.6%) for raw data and 85.4% (PC1 71.4%, PC2 14.0%) for adjusted data explained. With adequate results, the effect of retention time adjustment was also visualized in PCA in a previous study [60].

### 2.5. Multivariate Analysis and Phylogenetic Relationships

**Figure 5:**
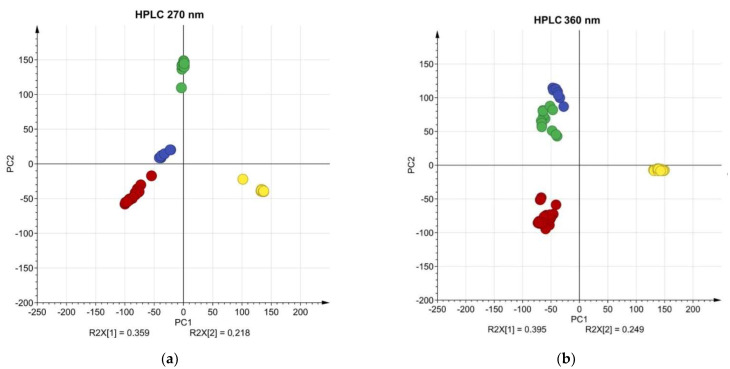
PCA clusters for analyzed medicinal plants. (**a**) PCA clusters for raw HPLC data detected at 270 nm (yellow—roselle, red—chamomile, green—yarrow, blue—calendula); (**b**) PCA clusters for raw HPLC data detected at 360 nm (yellow—roselle, red—chamomile, green—yarrow, blue—calendula).

To further investigate the differences of obtained HPLC chemical profiles, unsupervised multivariate analysis methods like PCA and HCA on raw HPLC data were executed. All chromatograms were normalized beforehand. All investigated medicinal plants in PCA formed tight clusters both at 360 nm and 270 nm wavelengths, respectively (Figure 5). At 360 nm the clusters of calendula and yarrow samples are grouped almost together, while at 270 nm the differentiation of clusters is superior, and all clusters are separate (Figure 5).

**Figure 6:**
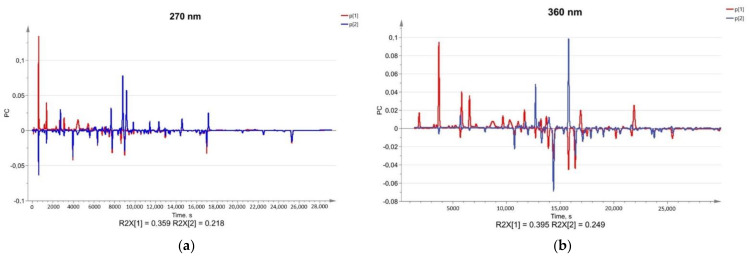
Loadings for HPLC methods. (**a**) Loadings for HPLC at 270 nm; (**b**) loadings for HPLC at 360 nm.

At 270 nm PC1 describes 35.9%, but PC2—21.8%, forming 57.7% of a total variance for the chromatographic information, while at 360 nm PC1 describes 39.5%, but PC2—24.9%, forming 64.4% of chromatographic information. A combination of the first ten components describes 95% (R2(X) 95.2%, Q2 92.4% at 270 nm, and R2(X) 95.7%, Q2 93.7% at 360 nm) of the composition of herbal compounds. The main differences in chemical composition are described by the PC1 and PC2, while later components have a smaller impact. The first two components at 270 nm describe a smaller portion of chromatographic information than at 360 nm, suggesting that at 270 nm more differences in chemical profiles were observed. PC1 can be used only to differentiate the roselle samples, while PC2 shows differences in the chemical composition of yarrow, calendula, and chamomile. Additionally, the loadings of PC2 at 270 nm wavelength describe more differences in chemical composition than at 360 nm, proving that data detection at 270 nm is superior for the differentiation of medicinal plants (Figure 6). From the PCA, we could see that the roselle cluster is the furthest from other clusters, suggesting its chemical composition is different to other investigated plants (Figure 5). 

**Figure 7:**
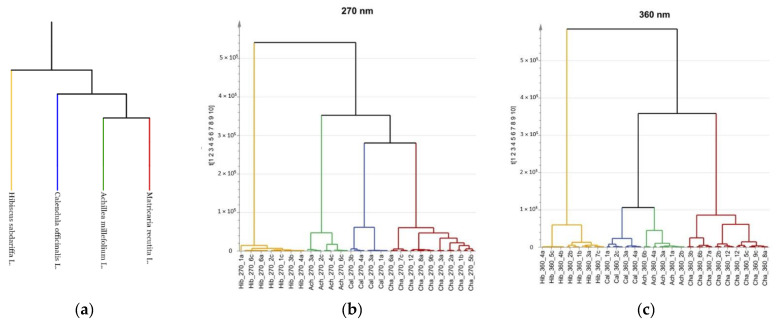
Correlation between phylogenetic relationships and fingerprint dataset groupings. (**a**) Phylogenetic tree of analyzed medicinal plants; (**b**) HCA dendrogram of HPLC data at 270 nm; (**c**) HCA dendrogram of HPLC data at 360 nm.

The clear difference in chemical composition of roselle samples was also expected based on the phylogenetic tree, a diagram that portrays the evolution of a set of species from their most recent common ancestor that was generated for these medicinal plants (Figure 7a) [61]. From the phylogenetic tree, it was observed that chamomile, calendula, and yarrow share a more recent common ancestor than either one share with roselle, suggesting that there will be a visible distinction of chemical profiles. Since the chemical composition is influenced also by the geographical origin of plant samples, and roselle samples came from tropical regions, but chamomile, calendula, and yarrow samples from Eastern Europe (mainly Latvia) the differences in chemical profiles could also be affected [6].

A correlation between the phylogenetic relationships and groupings of fingerprint datasets was also confirmed in HCA (Figure 7). Latvian native medicinal plant samples are located on one branch of the dendrogram, while roselle samples are on another branch, the same as in the phylogenetic tree. However a difference from the phylogenetic tree in HCA was seen for Latvian native medicinal plants (yarrow, calendula, and chamomile). In the phylogenetic tree, chamomile and yarrow share a more recent common ancestor than either one share with calendula (Figure 7a). That suggests that the chemical composition of yarrow and chamomile should be more alike. But the HCA proves otherwise—samples of chamomile are located closer to calendula samples in the dendrogram at 270 nm and samples of yarrow and calendula are grouped together at 360 nm, suggesting a more similar chemical composition, respectively (Figure 7). As previously mentioned, the chemical composition can be impacted by many factors, including the origin of samples, soil, growth conditions, and other reasons, which could have impacted this result [6]. Further research with larger sample sizes should be conducted to investigate this observation. In a study done by Kharyuk et al. a correlation was found between evolutionary relationships and herbal fingerprints for closely related species with samples made from similar parts, but groupings of higher order were not accurate [62]. The distance between clusters is larger at 270 nm, making the method superior for the identification of medicinal plants and for providing distinctive chemical profiles.

### 2.6. Application of the Herbal Fingerprinting Method

**Figure 8:**
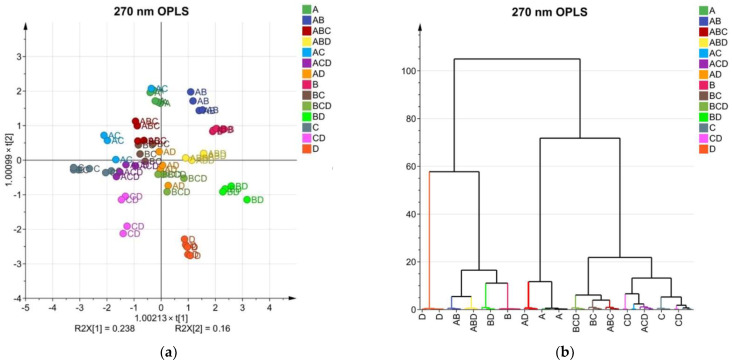

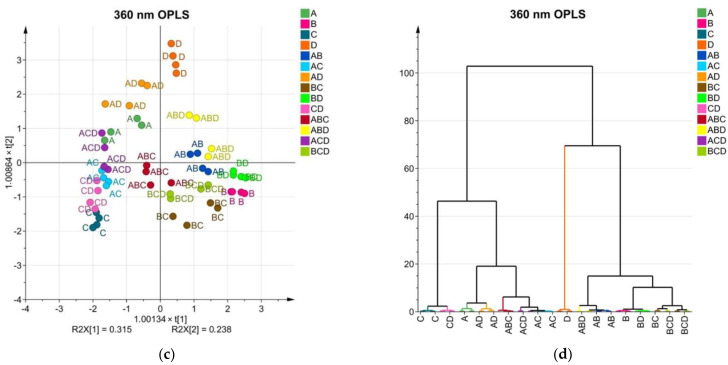
OPLS scatter plots and dendrograms for HPLC data of mixed extracts: (**a**) OPLS dendrogram of HPLC data of mixed extracts at 270 nm; (**b**) OPLS dendrogram of HPLC data of mixed extracts at 270 nm; (**c**) OPLS dendrogram of HPLC data of mixed extracts at 360 nm; (**d**) OPLS dendrogram of HPLC data of mixed extracts at 360 nm.

Mixed extracts of different combinations of the four analyzed medicinal plants were made, and HPLC, together with OPLS and HCA, was performed to see if we could identify the components of mixed extracts and to further investigate the possible application of the herbal fingerprinting method. OPLS has also been applied in previous studies to differentiate medicinal plants and their products [63,64,65]. The scatter plots and dendrograms of OPLS can be seen in Figure 8. Simple extracts made from one medicinal plant were also added to this analysis to see how the mixed extracts group in comparison to single medicinal plant extracts. Sets of simple and mixed extracts (56) were included in the OPLS analysis. OPLS analysis of data detected at 270 nm resulted in a tricomponent with R2(X) of 80.5%, R2(Y) of 91.4%, and Q2 of 88.5% of the variables. OPLS analysis of data detected at 360 nm resulted in tricomponent with R2(X) of 89.4%, R2(Y) of 83.2%, and Q2 of 80.7% of the variables. These results proved that the quality of the model was very good. From the scatter plots in Figure 8a,c we can see that the composition of mixed extracts influenced the location of clusters, correlated with the location of simple extracts, and indicated the components in the mixed extracts. For example, at 270 nm the clusters of mixed extract CD (chamomile and roselle) were located in between clusters of simple extracts—C (chamomile) and D (roselle). This tendency can be also seen in mixed extracts made from 3 different medicinal plants. For example, at 270 nm the clusters of mixed extract BCD (calendula, chamomile, and roselle) were located in between clusters of simple extracts—B (calendula), C (chamomile), and D (roselle). This observation can also be seen at 360 nm although the clusters are tighter and the separation of clusters is better at 270 nm. Dendrograms of OPLS (Figure 8b,d) show that samples tend to group according to their composition. At both wavelengths, roselle extract (D) is on a separate branch of the diagram with the highest Euclidean distance showing the dissimilarity from all other samples and the biggest difference in chemical composition. At 270 nm the dominant component is chamomile (C) because all mixed extracts containing chamomile tend to group closer to the simple chamomile extract than to other components of the mixed extract. For the same reason, at 360 nm the dominant component is calendula (B). OLPS analysis proves that the fingerprinting method can be used not only to identify extracts made from one medicinal plant but also to identify and characterize the composition of extracts made from several medicinal plants.

## 3. Materials and Methods

### 3.1. Plant Material

Twenty-five commercially available medicinal plant samples were collected from four different medicinal plants: roselle, chamomile, calendula, and yarrow. Chamomile, calendula, and yarrow were chosen because these medicinal plants are native to Latvia, are commonly used in Latvian traditional medicine, and all belong to the Asteraceae family. Roselle was chosen as a non-native, taxonomically different (Malvaceae family) medicinal plant to compare the chemical composition and fingerprints to the phylogenetic relationships of native and non-native medicinal plants, as well as to see if developed chromatographic methods can be used for a wider spectrum of medicinal plants. The detailed sample information is listed in Table 3. 

### 3.2. Chemicals and Reagents

Certified reference materials (CRM): caffeic acid (≥98%), apigenin (≥95%), and apigenin-7-glucoside were purchased from Sigma-Aldrich (St. Louis, MO, USA). Primary reference standards: rutin was purchased from PhytoLab (Vestenbergsgreuth, Germany), chlorogenic acid was purchased from the HWI group (Rülzheim, Germany). All solvents used were of analytical or HPLC grade. Water was distilled and purified using the Stakpure GmpH water system (Niederahr, Germany).

### 3.3. Sample Preparation

All medicinal plant samples were ground to a fine powder, and the powders were stored in airtight packaging at room temperature till extraction. 

To make a simple extract from only one medicinal plant 5.0 g of powdered medicinal plant sample was weighed and transferred to a 50 mL conical flask with a glass stopper, and 50 mL of 96% *v*/*v* ethanol was added. This procedure was executed in triplicate. Three extractions from each medicinal plant sample were made to reduce the impact of sample preparation and short time stability, as well as to additionally control measurement quality. The material was extracted by maceration for 24 h at room temperature. The extracts were filtered through Sartorius smooth filter paper, grade 3-HW (Göttingen, Germany), and the supernatant was collected in an airtight container and stored at 4 °C until chromatographic analysis. 

To test the application of our methods, mixed extracts from different combinations of analyzed medicinal plants were made. All combinations and their codes can be found in Table 4. Two different mixed extract preparation methods were executed—mixing together extracts already made from one medicinal plant (first method) and executing extraction from a mix of dry medicinal plant samples (second method). All combinations found in Table 4 were made using both methods.

In the first method, two simple extracts (ratio 50:50 *v*/*v*) or three simple extracts (ratio 33:33:33 *v*/*v*) were taken and transferred into airtight containers and carefully mixed. This procedure was executed in duplicate for all mixed extracts to reduce the impact of sample preparation. These extracts also were stored at 4 °C until chromatographic analysis.

In the second method, each needed medicinal plant sample was weighed and transferred to a 50 mL conical flask with a glass stopper, all dry samples were mixed together and ethanol 96% *v/v* was added with a ratio 1.0 g of mixed plant material: 10 mL ethanol. This procedure was executed in duplicate for all mixed extracts to reduce the impact of sample preparation. The materials were extracted by maceration for 24 h at room temperature. The extracts were filtered through Sartorius smooth filter paper, grade 3-HW (Göttingen, Germany), and the supernatant was collected in an airtight container and stored at 4 °C until chromatographic analysis.

The same certified reference materials and medicinal plant samples were used for the HPLC and TLC methods.

### 3.4. High-Performance Liquid Chromatography (HPLC) Conditions, Sample Preparation and Data Processing

HPLC analysis was performed on a DIONEX UltiMate 3000 UHPLC+ focused system (Thermo Fisher Scientific, Waltham, MA, USA), which consists of an UltiMate 3000 pump, UltiMate 3000 autosampler, UltiMate 3000 column compartment, UltiMate 3000 variable wavelength detector, and Chromeleon software. As a stationary phase, an Ascentis Express 90 Å AQ-C18 column (15 cm × 3.0 mm, 2.7 μm, Supelco, Darmstadt, Germany) was used. The mobile phase was composed of 0.1% aqueous trichloroacetic acid *(v*/*v)* (A) and acetonitrile (B) with the following gradient elution: 0 min—95% A, 45 min—55% A, 50 min—95% A. Before each sample analysis, a 4 min equilibration with 5% B was performed. The flow rate was set at 0.425 mL/min, the column temperature was 40 °C, injection volume was 1 μL, and the time of analysis was 50 min. The detection wavelength was set at 360 nm and 270 nm. Before HPLC analysis, extracts were put at room temperature for 2 h to reach room temperature and reduce the possibility of sample temperature influence on results. Before analysis, extracts were filtered through syringe filters with 0.45 μm pore size. Peaks with early retention time (<4 min) were omitted from the chemical profiles and further analysis due to the chance of errors and false signals not related to the composition of the sample. The average chromatogram for each medicinal plant was generated by combining all obtained chromatograms according to detection wavelengths. Since the quantitative concentrations of compounds were not investigated, all chromatograms were normalized beforehand. HPLC was performed on both the simple extracts made from one medicinal plant and mixed extracts.

### 3.5. Thin Layer Chromatography (TLC) Conditions and Sample Preparation

TLC analysis was performed on a CAMAG TLC system (Muttenz, Switzerland) and visionCATS software. HPTLC Silica gel 60 F254 glass plates 20 cm × 10 cm from Merck (Darmstadt, Germany) were used as a stationary phase. The plates were activated at 60 °C for one hour before use. Before TLC analysis, extracts were put at room temperature for 2 h. Using the CAMAG Linomat 5 semi-automatic sampler, 7 μL samples were applied using a 100 μL syringe. Samples were applied with 8 mm bandwidth and 8 mm from the bottom of the plate. The number of samples on each plate differed between medicinal plants. The syringe was washed with purified water between each sample. The CAMAG ADC2 automatic developing chamber was saturated with the mobile phase ethyl acetate:water:formic acid 80:10:10 (*v*/*v*/*v*) for 30 min and then developed until 80 mm height. The same mobile phase has been used in two previous studies analyzing *Salvia officinalis* L. extracts and *Artemisia* species, and it is also stated as the TLC method for analyzing Calendula flowers by the European Pharmacopoeia [39,66,67]. After development, the plates were visualized in the TLC Visualizer 2 under UV wavelengths of 366 nm, 254 nm, and white light. TLC was performed only on simple extracts made from one medicinal plant.

### 3.6. Phylogenetic Tree

A phylogenetic tree was constructed using the PhyloT v2 platform based on the NCBI taxonomy [68]. The constructed phylogenetic tree was visualized using the iTOL platform [69]. 

### 3.7. Data Analysis

All experiments were performed at least in triplicate with constant results. Differences among groups were considered significant at *p* < 0.05. R_f_ values in thin layer chromatography were measured using an R_f_ value tool provided by the visionCATS software. This software was also used to automatically generate profiles of analyzed medicinal plants. The raw HPLC chromatographic data were exported as *.txt format files. Chromatogram normalization and visualization were conducted, and the average chromatogram for each plant was generated on SpectraGryph 1.2.15. software (Friedrich Menges, Oberstdorf, Germany) [70]. SpecAlign software was used to adjust HPLC retention times to the plant’s average chromatogram. The used method was first proposed by J.W.H. Wong et al. and released in SpecAlign 2.4.1 (University of Oxford, Oxford, United Kingdom) [58]. PCA, HCA, and OPLS were performed using SIMCA 14 software (Umetrics, Umea, Sweden). The formation of clusters was visualized in scatter plots, dendrograms, and loadings. HCA was calculated using Ward’s algorithm. Pearson’s correlation coefficients were calculated on Origin 10 software (Originlab, North Hampton, MA, USA). PCA, HCA, OPLS and similarity analysis were carried out on both the HPLC raw data and HPLC data with adjusted retention times. 

## 4. Conclusions

The herbal fingerprinting method has a great potential for the identification and characterization of herbal extracts. Research shows clear differences in chemical fingerprints of Latvian native medicinal plants (chamomile, calendula, and yarrow) and roselle. TLC can be used as an initial screening tool of herbal extracts, while HPLC gives much more detailed information regarding the chemical composition of herbal extracts. HPLC fingerprint similarity can be greatly improved by adjusting retention times to the average chromatogram of each medicinal plant. In HPLC data, detection at 270 nm is superior to 360 nm and provides more detailed results of the chemical composition of herbal extracts and can be used to identify and differentiate medicinal plants. The correlation between the phylogenetic relationships and groupings of fingerprint datasets of analyzed medicinal plants was confirmed. The developed HPLC method in conjunction with chemometrics can also be used to identify components of mixed extracts containing different combinations of analyzed medicinal plants. Future research using other methods such as mass-spectrometry and larger sample sizes should be conducted to gain a deeper knowledge of the chemical composition of herbal extracts.

## Figures and Tables

**Table 1:** Identified chemical compounds and their retention times.

Wavelength	Chemical Compound	Standard RT (min)	Sample RT ^1^ (min)	Yarrow	Chamomile	Calendula	Roselle
270 nm	Chlorogenic acid	9.86	9.85 ± 0.04	+	+	+	+
	Caffeic acid	10.37	10.37 ± 0.01	+	+		+
	Rutin	17.99	17.99 ± 0.01	+		+	+
	Apigenin-7-glucoside	21.41	21.42 ± 0.01	+	+		
	Apigenin	32.34	32.36 ± 0.05	+	+		
360 nm	Chlorogenic acid	9.87	9.88 ± 0.02	+	+	+	+
	Caffeic acid	10.37	10.39 ± 0.05	+	+		+
	Rutin	17.99	17.99 ± 0.01	+		+	+
	Apigenin-7-glucoside	21.42	21.42 ± 0.02	+	+		
	Apigenin	32.37	32.37 ± 0.05	+	+		

^1^ Results are shown as average RT value ± RT variance between samples. “+” chemical compound has been identified in ethanol extracts.

**Table 2:** Pearson’s correlation coefficient values for raw data and data with adjusted retention times.

Medicinal Plant	Average Pearson’s Correlation Coefficient Value (270 nm)		Average Pearson’s Correlation Coefficient Value (360 nm)	
	Raw Data	Data with Adjusted Retention Times	Raw Data	Data with Adjusted Retention Times
Roselle	0.9628	0.9764	0.8519	0.9648
Chamomile	0.7122	0.7995	0.7220	0.8116
Calendula	0.7997	0.9457	0.8909	0.9762
Yarrow	0.6805	0.9305	0.8191	0.9305

**Table 3:** Used medicinal plant samples.

Medicinal Plant	Number of Samples	Sample Origin Country/Region	Code in Figures
Roselle (*Hibiscus sabdariffa* L.)	7	Africa (5)	Hib or D
		Uzbekistan (1)	
		Jamaica (1)	
Chamomile (*Matricaria recutita* L.)	9	Latvia (7)	Cha or C
		Poland (2)	
Calendula *(Calendula officinalis* L.)	4	Latvia (4)	Cal or B
Yarrow *(Achillea millefolium* L.)	5	Latvia (5)	Ach or A

**Table 4:** Combinations of medicinal plants in mixed extracts.

Medicinal Plants	Code in Figures
Yarrow and calendula	AB
Yarrow and chamomile	AC
Yarrow and roselle	AD
Calendula and chamomile	BC
Calendula and roselle	BD
Chamomile and roselle	CD
Yarrow, calendula, chamomile	ABC
Yarrow, calendula, roselle	ABD
Yarrow, chamomile, roselle	ACD
Calendula, chamomile, roselle	BCD

## Data Availability

Data is contained within the article.

## Supplementary Materials

The following supporting information can be downloaded at: https://www.mdpi.com/article/10.3390/molecules27082555/s1. Figure S1: Developed HPTLC plate of *Achillea millefolium* L. extracts under 366 nm UV light. Figure S2: Developed HPTLC plate of *Matricaria recutita* L. extracts and chlorogenic acid under 366 nm UV light. Figure S3: Developed HPTLC plate of *Calendula officinalis* L. extracts under 366 nm UV light. Figure S4: Developed HPTLC plate of *Hibiscus sabdariffa* L. extracts and rutin under 366 nm UV light. Figure S5: The average HPLC chromatogram of *Hibiscus sabdariffa* L., 270 nm. Figure S6: The average HPLC chromatogram of *Calendula officinalis* L., 270 nm. Figure S7: The average HPLC chromatogram of *Matricaria recutita* L., 270 nm. Figure S8: The average HPLC chromatogram of *Achillea millefolium* L., 270 nm. Figure S9: The average HPLC chromatogram of *Hibiscus sabdariffa* L., 360 nm. Figure S10: The average HPLC chromatogram of *Calendula officinalis* L., 360 nm. Figure S11: The average HPLC chromatogram of *Matricaria recutita* L., 360 nm. Figure S12: The average HPLC chromatogram of *Achillea millefolium* L., 360 nm.

## Sample Availability

Samples are not available.
