# Supplementary material for: The Potential Use of Herbal Fingerprints by Means of HPLC and TLC for Characterization and Identification of Herbal Extracts and the Distinction of Latvian Native Medicinal Plants
Source: Molecules. 2022 Apr 15;27(8):2555. doi: 10.3390/molecules27082555 (PMC9026908; doi:10.3390/molecules27082555)
Supplement: Supplementary file 1 [file molecules-27-02555-s001.zip › molecules-1653548-supplementary.pdf]

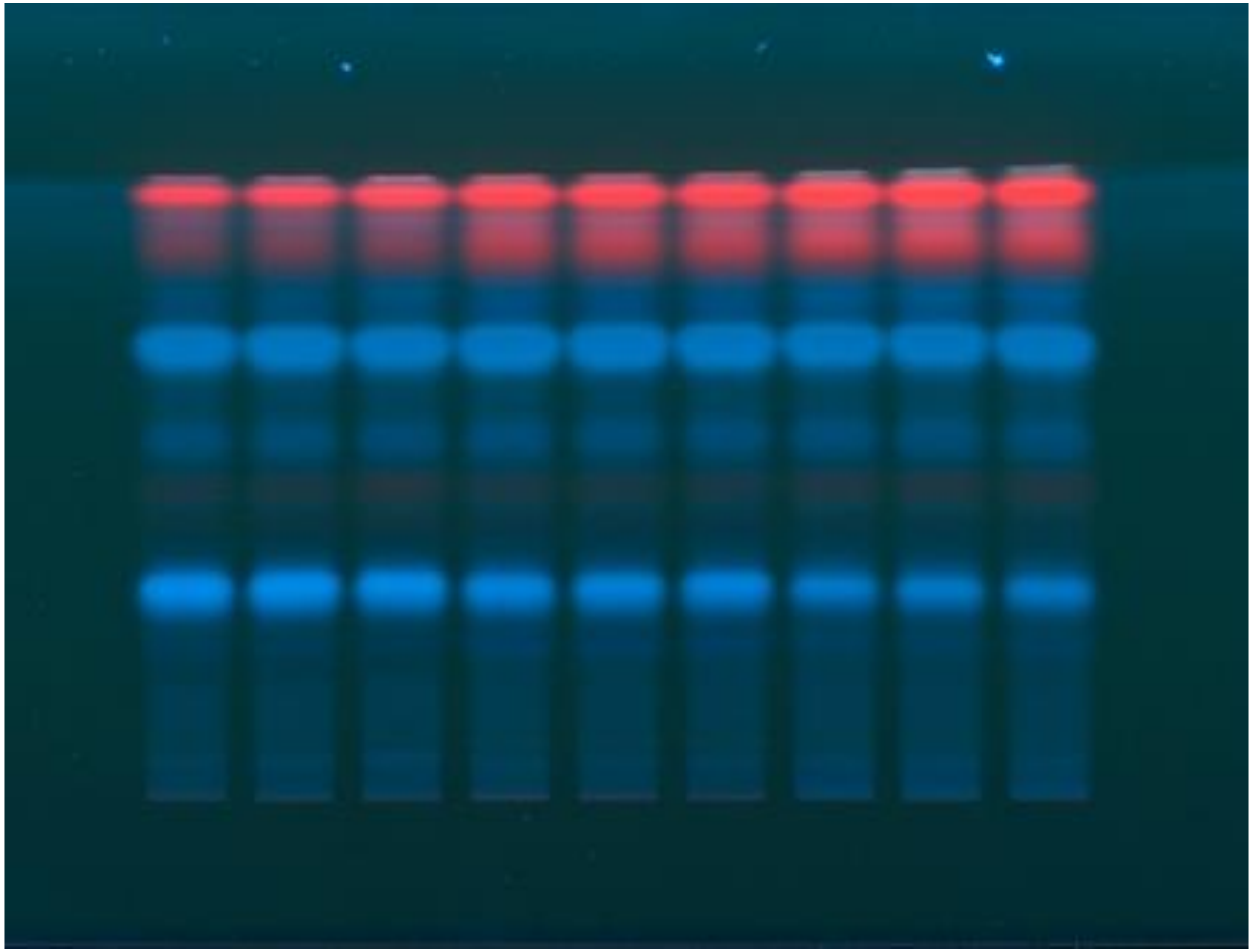

Figure S1. Developed HPTLC plate of *Achillea millefolium* L. extracts under 366 nm UV light.

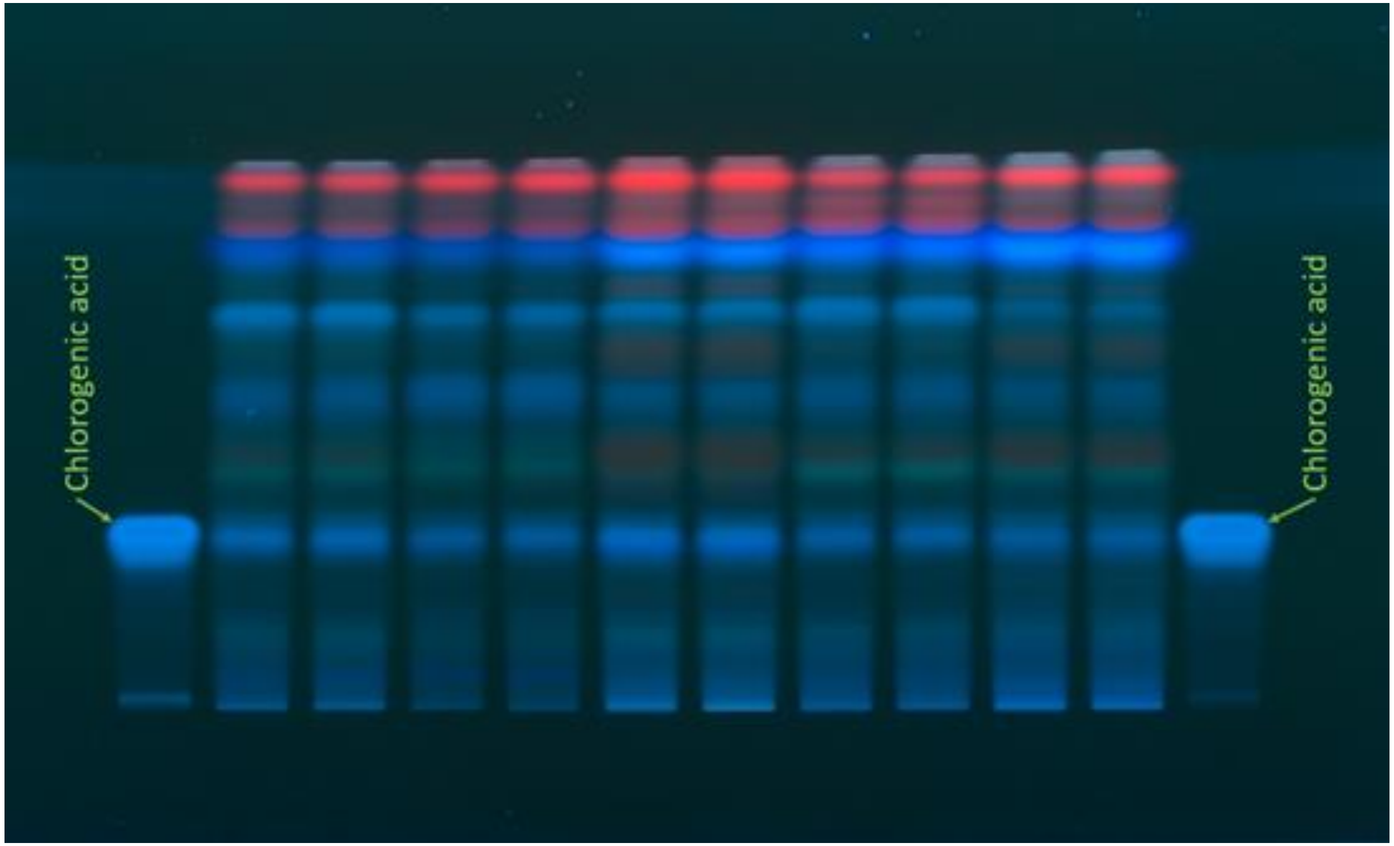

Figure S2. Developed HPTLC plate of *Matricaria recutita* L. extracts and chlorogenic acid under 366 nm UV light.

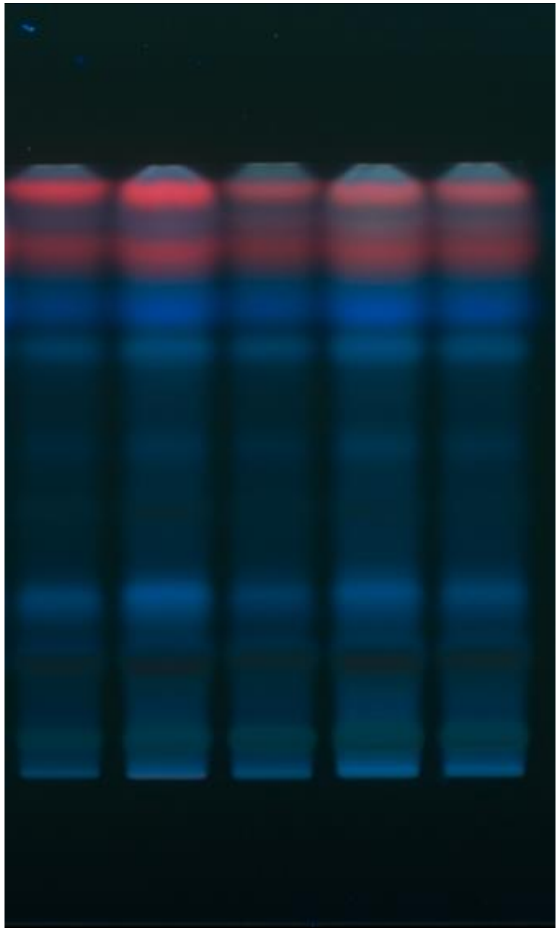

Figure S3. Developed HPTLC plate of *Calendula officinalis* L. extracts under 366 nm UV light.

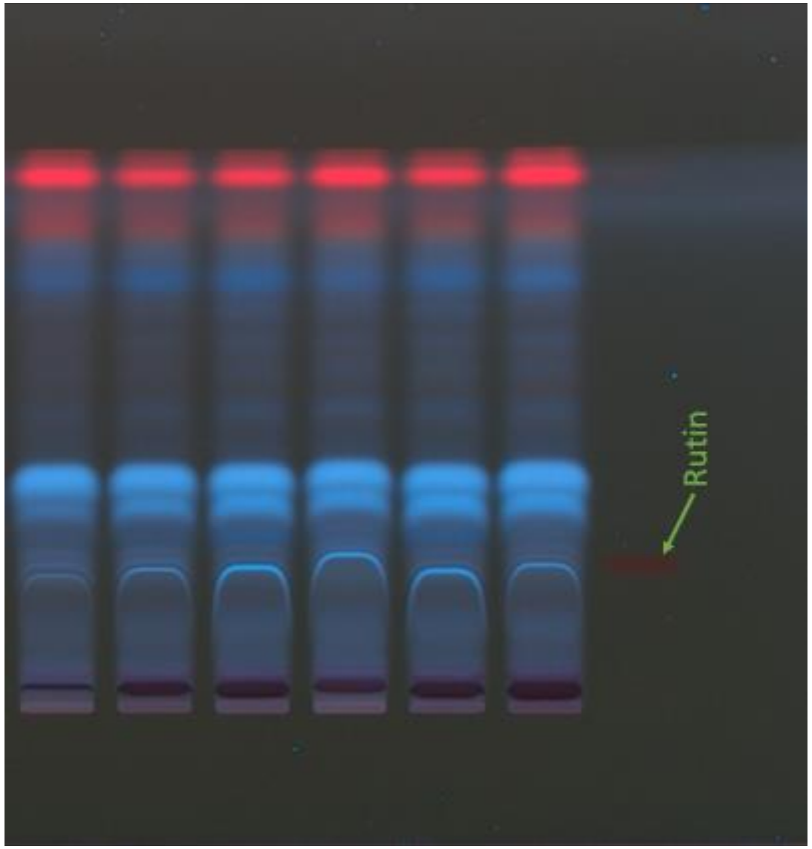

Figure S4. Developed HPTLC plate of *Hibiscus sabdariffa* L. extracts and rutin under 366 nm UV light.

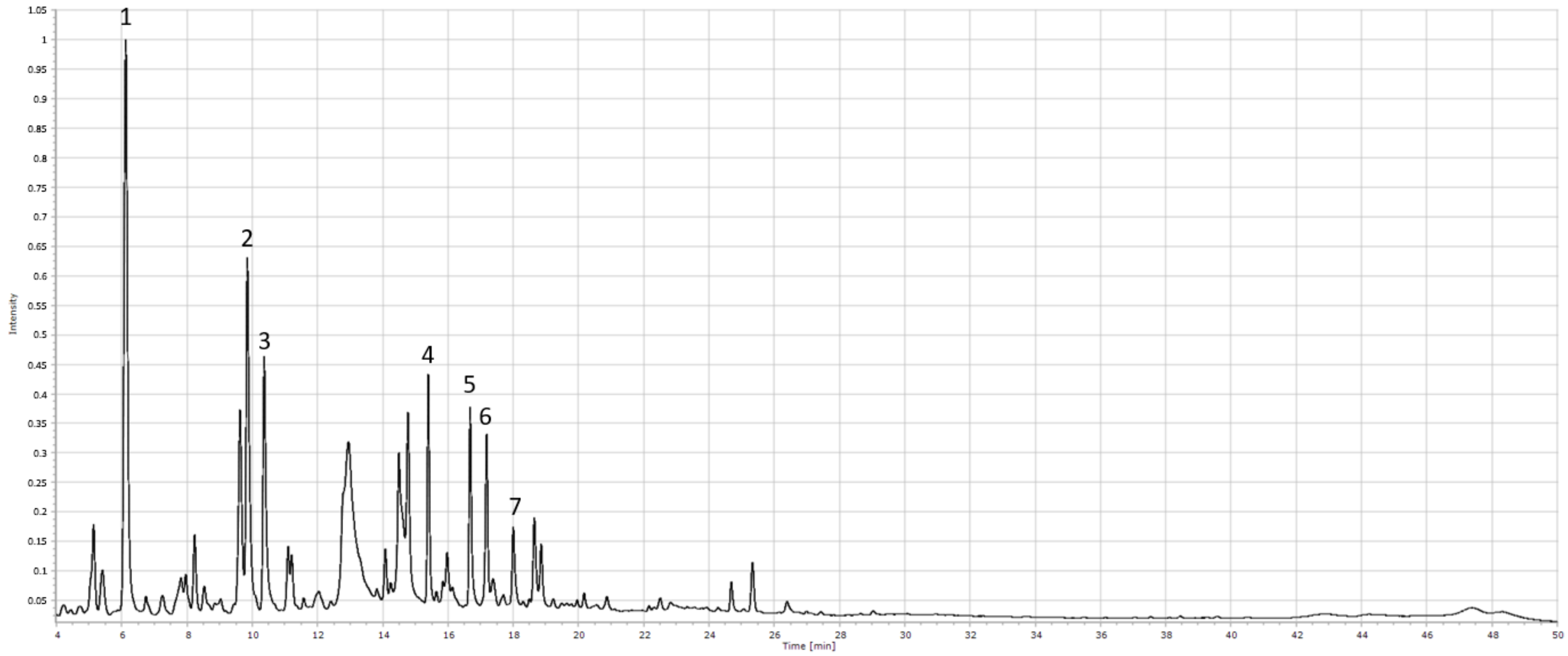

Figure S5. The average HPLC chromatogram of *Hibiscus sabdariffa* L. extracts, 270 nm. 2 – chlorogenic acid; 3 – caffeic acid; 7 – rutin.

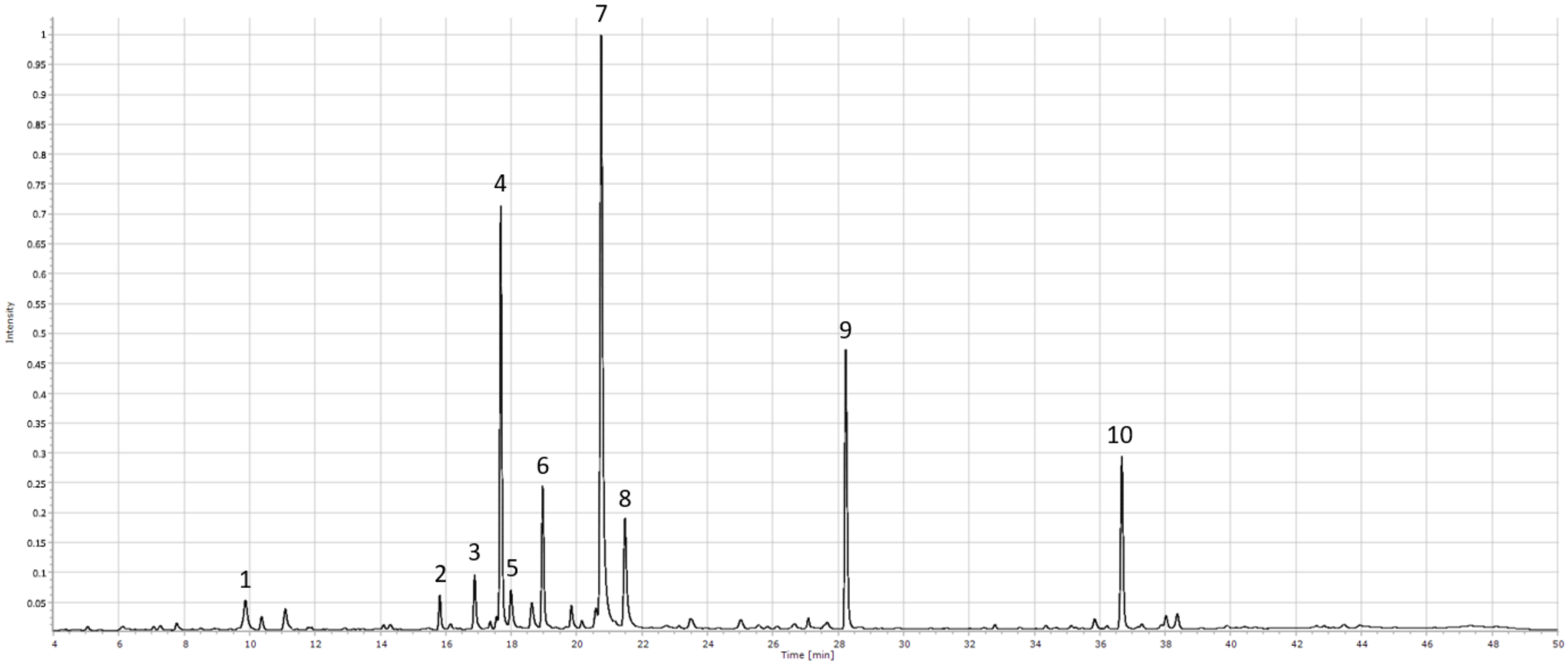

Figure S6. The average HPLC chromatogram of *Calendula officinalis* L. extracts, 270 nm. 1 – chlorogenic acid; 5– rutin.

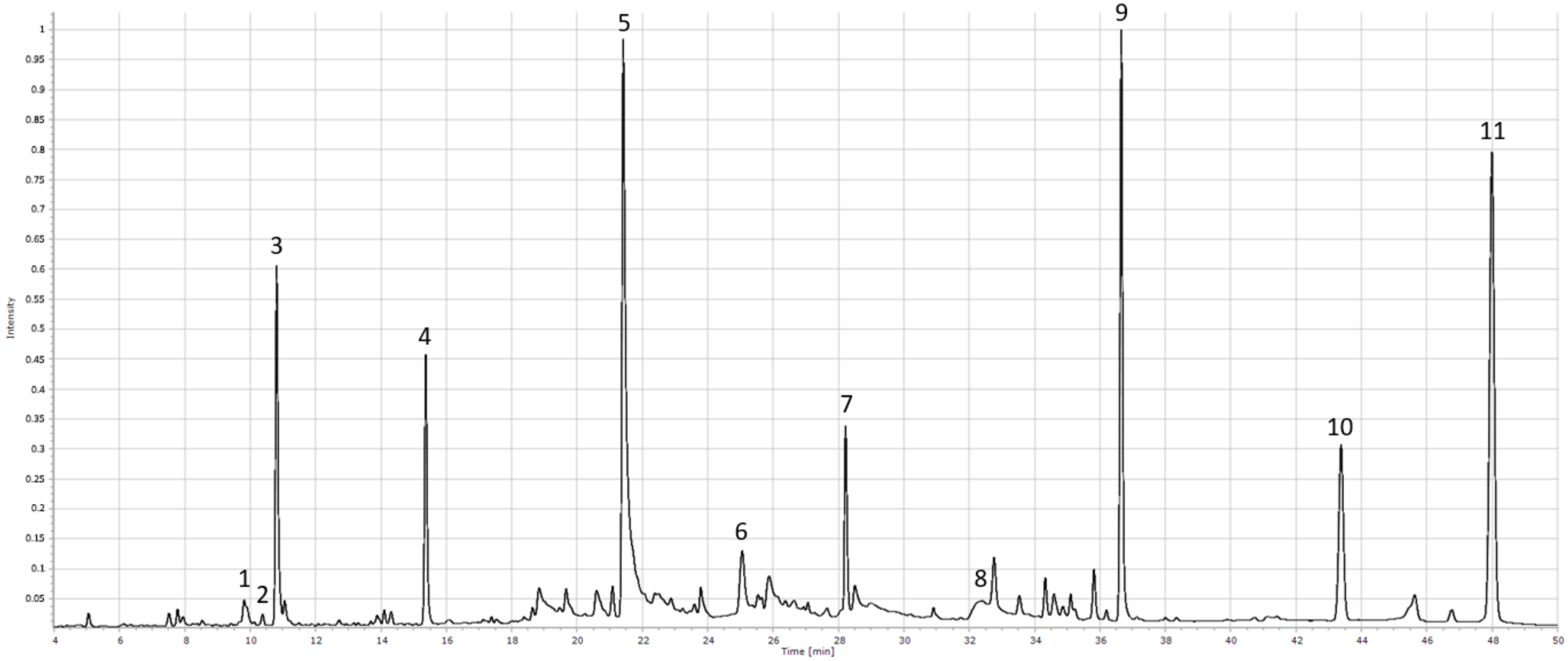

Figure S7. The average HPLC chromatogram of *Matricaria recutita* L. extracts, 270 nm. 1 – chlorogenic acid; 2 – caffeic acid; 5 – apigenin-7-glucoside; 8 – apigenin.

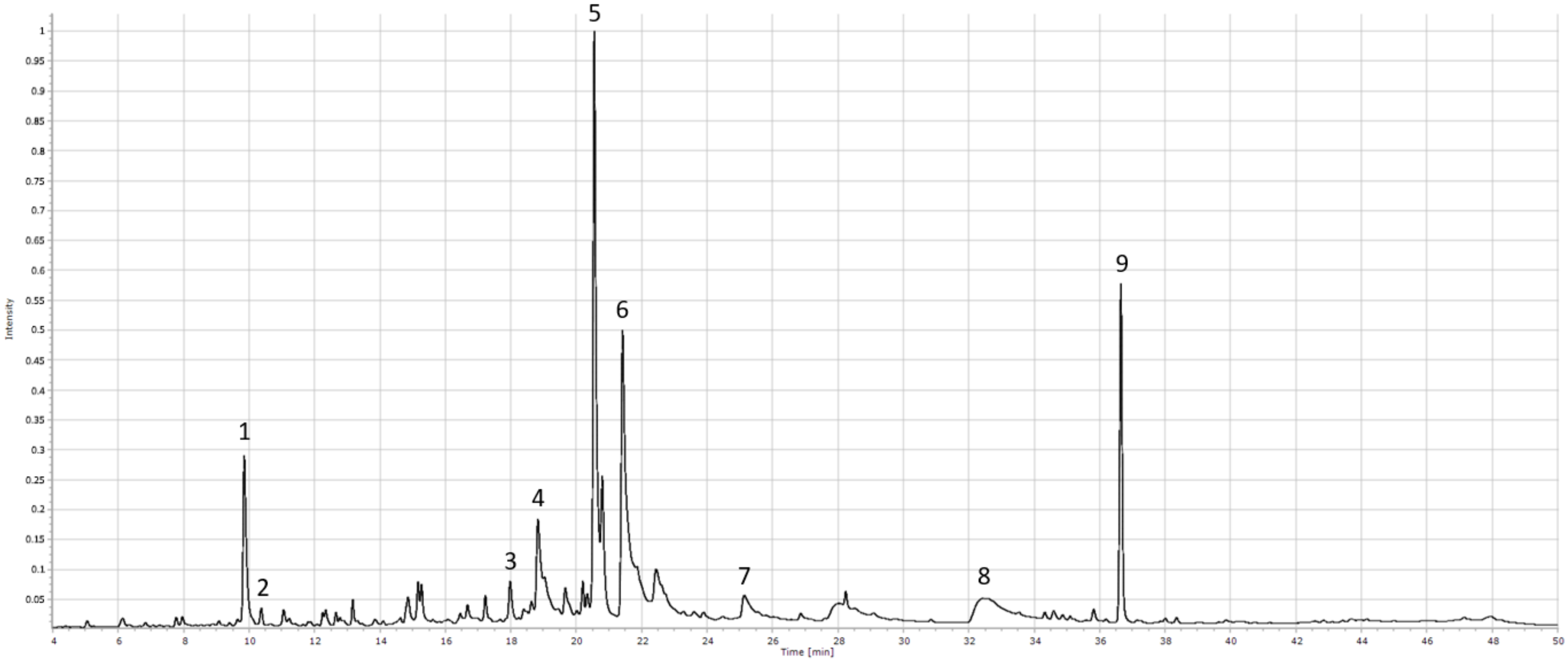

Figure S8. The average HPLC chromatogram of *Achillea millefolium* L. extracts, 270 nm. 1 – chlorogenic acid; 2 – caffeic acid; 3 – rutin; 6 – apigenin-7-glucoside; 8 – apigenin.

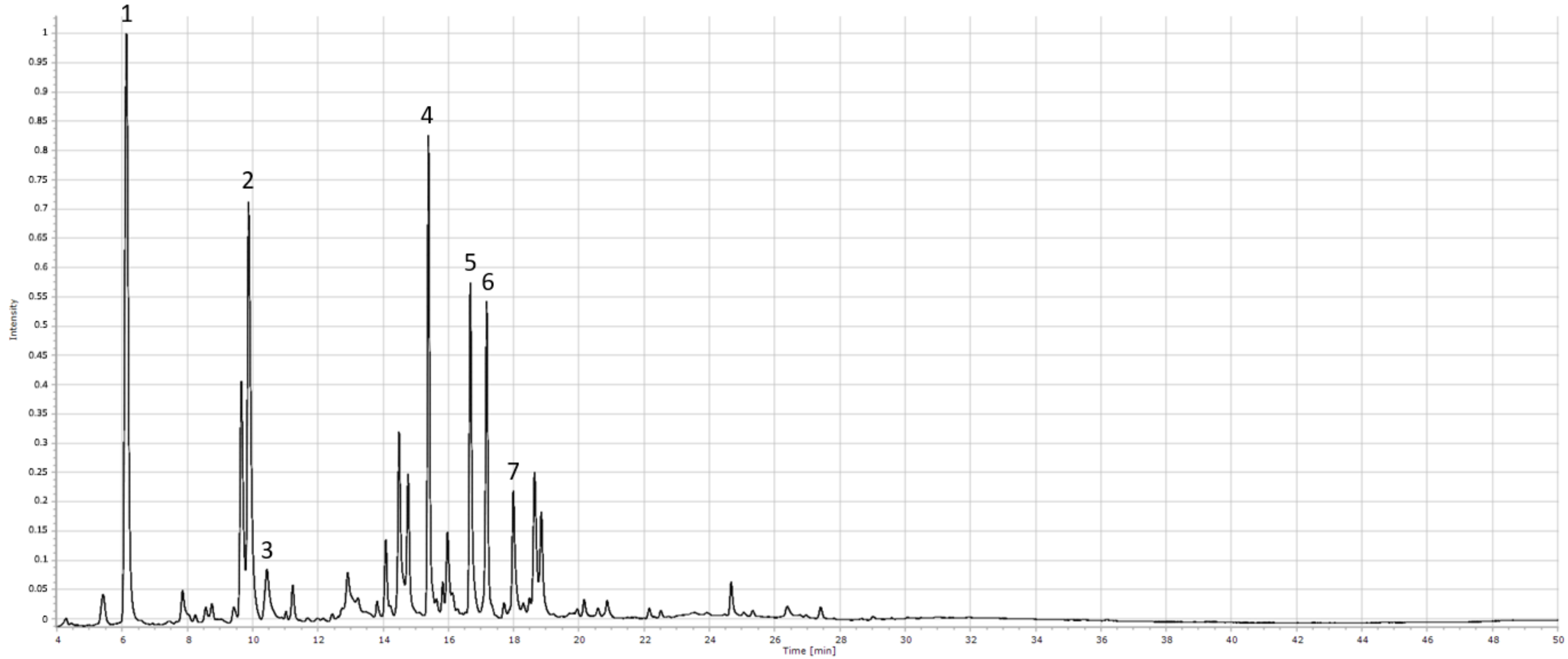

Figure S9. The average HPLC chromatogram of *Hibiscus sabdariffa* L. extracts, 360 nm. 2 – chlorogenic acid; 3 – caffeic acid; 7 – rutin.

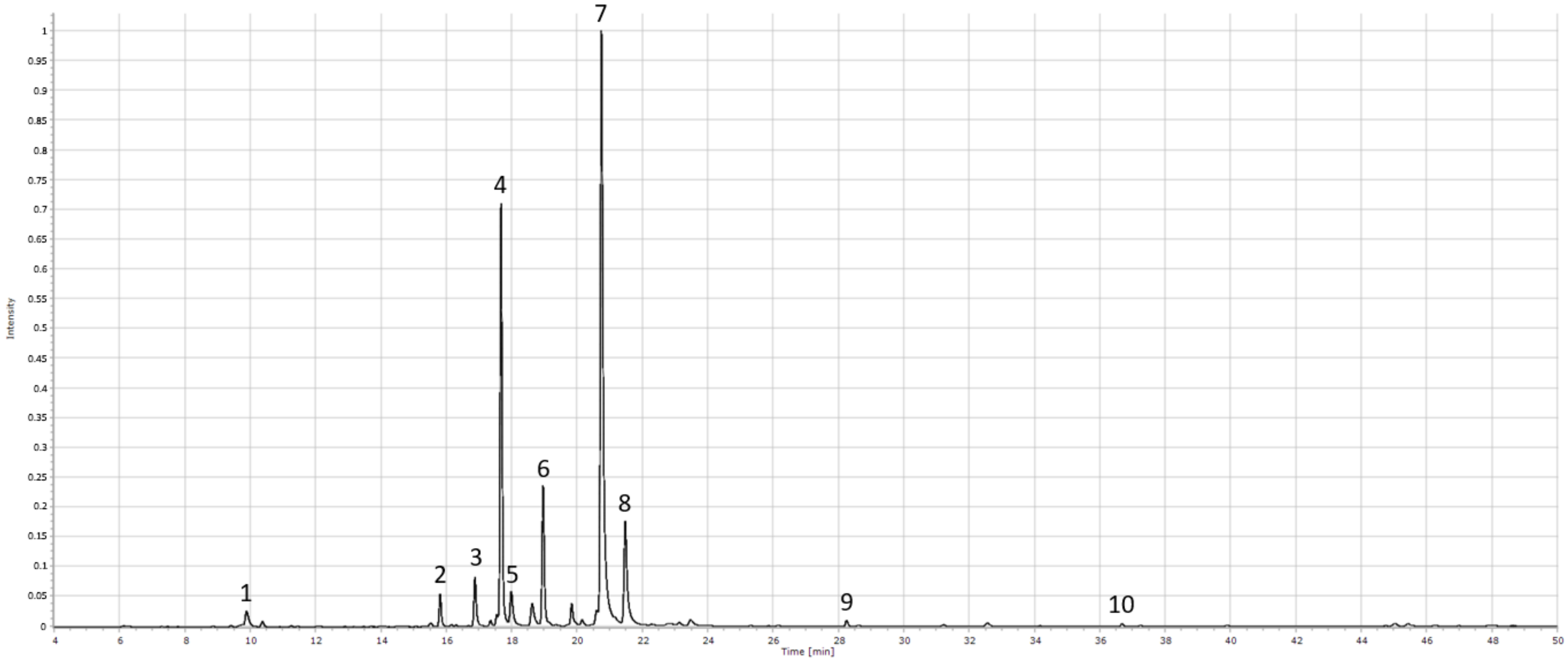

Figure S10. The average HPLC chromatogram of *Calendula officinalis* L. extracts, 360 nm. 1 – chlorogenic acid; 5– rutin.

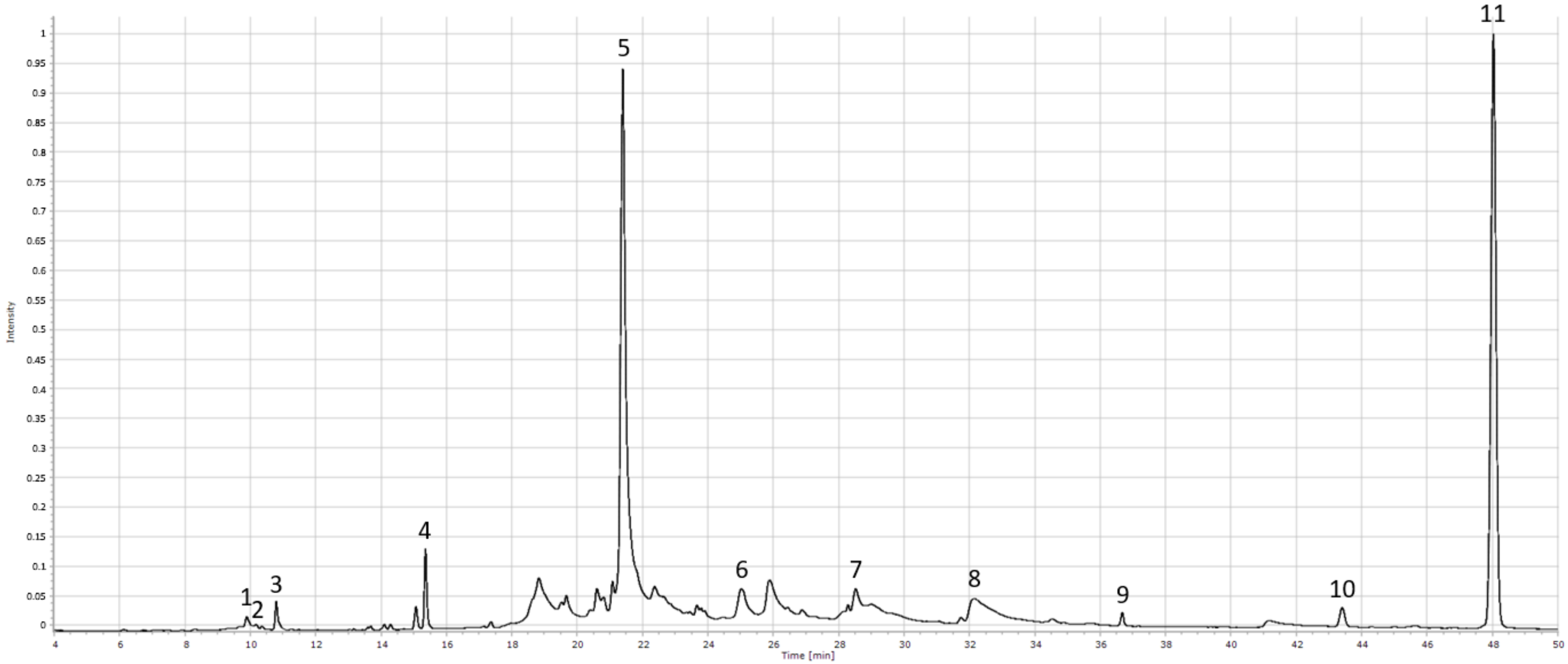

Figure S11. The average HPLC chromatogram of *Matricaria recutita* L. extracts, 360 nm. 1 – chlorogenic acid; 2 – caffeic acid; 5 – apigenin-7-glucoside; 8 – apigenin.

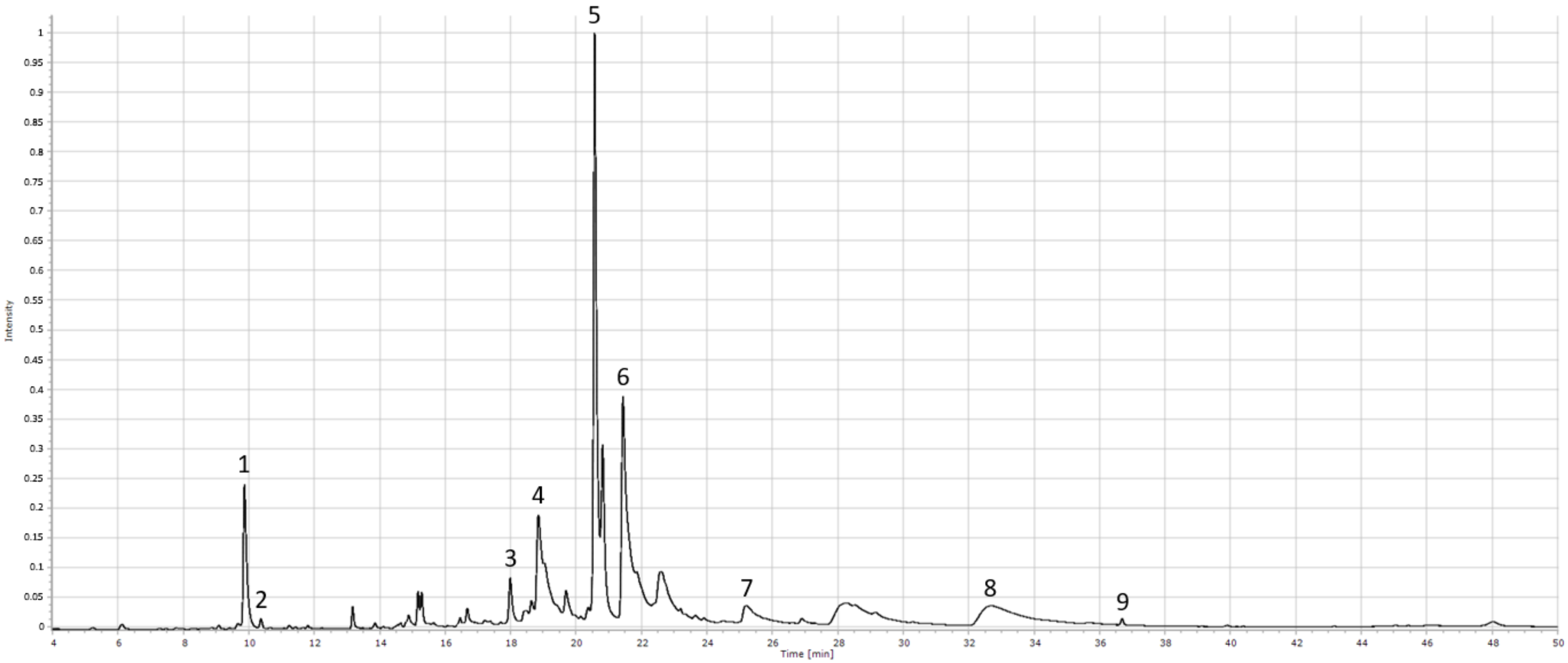

Figure S12. The average HPLC chromatogram of *Achillea millefolium* L. extracts, 360 nm. 1 – chlorogenic acid; 2 – caffeic acid; 3 – rutin; 6 – apigenin-7-glucoside; 8 – apigenin.
